# Hypervirulent *Klebsiella pneumoniae* (hv*Kp*): Overview, Epidemiology, and Laboratory Detection

**DOI:** 10.20411/pai.v10i1.777

**Published:** 2025-01-24

**Authors:** Dania Al Ismail, Edgar I. Campos-Madueno, Valentina Donà, Andrea Endimiani

**Affiliations:** 1 Institute for Infectious Diseases (IFIK), University of Bern, Bern, Switzerland; 2 Independent Researcher and Scientific Writer, Bolzano, Italy

**Keywords:** *Klebsiella pneumoniae*, virulence, hv*Kp*, epidemiology, detection, ESBL, carbapenemases, string test, hypermucoviscous, WGS, *in vivo*

## Abstract

*Klebsiella pneumoniae* (*Kp*) is a Gram-negative pathogen responsible for both hospital- and community-acquired infections. *Kp* is classified into 2 distinct pathotypes: classical *K. pneumoniae* (c*Kp*) and hypervirulent *K. pneumoniae* (hv*Kp*). First described in Taiwan in 1986, hv*Kp* are highly pathogenic and characterized by unique phenotypic and genotypic traits. The hypermucoviscous (hmv) phenotype, generally marked by overproduction of the capsule, is often associated with hv*Kp*, although recent studies show that some c*Kp* strains may also have this characteristic. Furthermore, hv*Kp* can cause severe community-acquired infections in healthy people and have been associated with metastatic infections such as liver abscess, meningitis, and endophthalmitis.

Hv*Kp* are increasingly being reported in hospital-acquired settings, complicating treatment strategies. In particular, while hv*Kp* have historically been antibiotic-susceptible, multidrug-resistant (MDR) strains have emerged and pose a significant public health threat. The combination of high virulence and limited antibiotic options demands further research into virulence mechanisms and rapid identification methods.

This review discusses the epidemiology of hv*Kp* and their virulence factors, highlighting the importance of phenotypic and non-phenotypic tests, including next-generation molecular diagnostics, for the early detection of hv*Kp*.

## INTRODUCTION

*Klebsiella pneumoniae* (*Kp*) is a well-known Gram-negative opportunistic pathogen within the *Enterobacteriaceae* family that is frequently responsible for both hospital- and community-acquired infections [1–3]. *Kp* is classified into 2 distinct pathotypes – classical *K. pneumoniae* (c*Kp*) and hypervirulent *K. pneumoniae* (hv*Kp*) – which vary in their clinical presentation, epidemiology, identification methods, and management strategies (Table 1) [4–6].

**Table 1.** General Characteristics of Hypervirulent and Classical *Klebsiella pneumoniae* Strains

Features	hv*Kp*	c*Kp*
Acquisition of infections	Community	Hospitals and longterm care facilities
Population affected	All ages, healthy individuals	Elderly, immunocompromised
Number of infection sites	Multiple	Single
Metastasis or additional site	Common	Uncommon
Liver abscess	Not associated with biliary disease	Associated with biliary disease
Unusual infectious	Endophthalmitis, meningitis, septic arthritis, splenic abscess, necrotizing fasciitis	Rare
Hmv phenotype	Usual	Rare
Geographic spread	Worldwide	Worldwide
Serotypes identified	Kl, K2, K5, K16, K20, K54, K57, KN1	Kl to K79
Siderophores production	Aerobactin, salmochelin, enterobactin, yersiniabactin	Enterobactin, yersiniabactin
Acquired virulence-encoding genes	*magA* (mucoviscosity-associated gene A), *rmpA*, *rmpA2* (regulators for capsule), *peg-344* (inner membrane transporter), *iucA*, *iutA* (aerobactin biosynthesis), *iroB* (salmochelin biosynthesis), *clb* (colibactin), *terW* (tellurite resistance)	May possess some of them [33]

**Note.** hv*Kp*, hypervirulent *K*. *pneumoniae*; c*Kp*, classical *K*. *pneumoniae*; hmv, hypermucoviscous; ICE, integrative conjugative element

Since its first description in 1986 in Taiwan [7], hv*Kp* has emerged worldwide as a particularly virulent pathotype with unique phenotypic and genotypic characteristics [8–10]. For instance, a trait that is usually associated with hv*Kp* is a hypermucoviscous (hmv) phenotype with an overproduction of the capsule [11–13]. However, recent studies have found that not all hv*Kp* strains are hmv and some c*Kp* strains may also possess this characteristic [4, 5, 14, 15].

While c*Kp* is a frequent cause of primary pneumonia and urinary tract infections in the hospital setting (especially in the elderly or immunocompromised patients [16, 17]), hv*Kp* is more virulent and can cause community-acquired infections in healthy individuals, even though hospital-acquired infections are being increasingly reported as well [4, 18]. Besides its role as a main causative agent of pyogenic liver abscess [19], hv*Kp* may also lead to other unusual and multiple body-site infections (ie, metastatic spread), such as endophthalmitis, meningitis, septic arthritis, lung abscess, pneumonia, epidural abscess, osteomyelitis, non-hepatic abscesses, and necrotizing fasciitis [9, 11, 15, 18, 20–24]. Overall, infections due to hv*Kp* have high rates of morbidity and mortality [25–27]. Therefore, prompt therapeutic interventions are essential to prevent a poor prognosis [2, 15, 28].

The prevalence of the hypervirulent pathotype amongst *Kp* is quite variable, but it may be as high as 12% to 45% in endemic areas like China [29–31]. Complicating matters is the fact that, in the past, hv*Kp* isolates were primarily susceptible to antibiotics, but many recent studies report the emergence of multidrug-resistant (MDR) strains [32]. As a consequence, the combination of high virulence and limited antibiotic treatment options represents an ultimate threat to our public health systems. This concerning fact has spurred increased research attention towards this pathogen, focusing on identifying its main virulence markers. Therefore, understanding the virulence mechanisms underlying this pathotype and its rapid identification are crucial for the development of effective treatments and preventive measures.

This review provides an overview of the epidemiology of hv*Kp* and underscores the importance of investigating their virulence factors (VFs). Furthermore, we explore laboratory detection methods – with particular emphasis on molecular diagnostics – to facilitate rapid identification of infections caused by hv*Kp* strains.

## HYPERVIRULENCE FACTORS

Factors that may contribute to *Kp* hypervirulence, such as capsular hyperproduction, hmv phenotype, siderophores, and other potential contributors (eg, lipopolysaccharides (LPS), colibactin and fimbriae) have been extensively studied since its first emergence 4 decades ago. So far, several biomarkers associated with hypervirulence have been identified more frequently in hv*Kp* than c*Kp* strains (Table 1), including genetic components on chromosomes, virulence plasmids, or a combination of both [27, 34]. Of note, most of the main VFs of hv*Kp* are usually located on plasmids or within mobile genetic elements (MGEs) integrated in the chromosome, suggesting that horizontal gene transfer (HGT) may be an important mechanism for the development of a hypervirulent phenotype [35].

### Capsule (Over)production

The production of peculiar capsular polysaccharides is a crucial factor for *Kp* survival within the host and represents a key virulence determinant for its capacity to evade phagocytosis, complement, antimicrobial peptides, and specific antibodies [2, 18, 36–38]. In this context, the serological classification of *Kp* typically relies on capsule (ie, K antigen) serotyping, with at least 79 distinct capsule types identified to date [33, 39–41]. In particular, 8 types (K1, K2, K5, K16, K20, K54, K57, and KN1) have been described in hv*Kp* (Table 1) [36, 42–44], with K1 and K2 being the most frequently reported [18, 27, 36, 45].

Basic capsule production in *Kp* is a process regulated by genes located in the capsule polysaccharide synthesis (*cps*) locus of the chromosome, including genes such as *wzi*, *wza*, *wzb*, *wzc*, *gnd*, *wca*, *cpsB*, *cpsG*, and *galF* [46].

Capsule overproduction is influenced by several chromosomal genes, such as the mucoviscosity-associated gene A (*magA*; newly termed as *wzy-K1*, ie, the serotype K1 polymerase gene in the *cps* locus) and both _c_*rmpA* and _c_*rmpA2* genes (encoding regulators of the mucoid phenotype). Moreover, capsule overproduction can also be influenced by the plasmid-mediated genes _p_*rmpA*, p*rmpA2*, and *peg-344* (encoding a putative transporter; see below) that can be found with different frequencies on virulence plasmids (eg, pLVPK with all 3 genes; pVir-CR-hvKp4 with only _p_*rmpA2*) [11, 46]. Interestingly, *rmpA* and *peg-344* can also be found in the integrative conjugative element (ICE) of serotype K1 *Kp* (ICE*Kp1*)*.* Overall, the above chromosomal or plasmidic genes are regarded as valid molecular markers for the detection of hv*Kp*, although c*Kp* may also rarely possess some of them (Table 1) [15, 22, 27, 33, 36, 47–49]. Particularly, *rmpA* is considered specific for hv*Kp* identification due to its significant contribution in hypercapsule production, enhancing the pathogenicity of hv*Kp* [11, 48].

### Hypermucoviscous Phenotype

The most striking feature of the majority of hv*Kp* is the hypermucoviscous (hmv) phenotype, a trait that is usually associated with excess capsule synthesis (see above). However, the real link between hmv phenotype and capsule overproduction is still under investigation.

Indeed, the hmv phenotype is not solely determined by capsular polysaccharide overproduction, but is also associated with additional factors, such as the presence of *rmpC* and *rmpD* genes (ie, capsular and hmv regulators, respectively) located on the virulence plasmids [50, 51]. In this context, a Δ*rmpC Kp* mutant was shown to retain hypermucoviscosity but produced less capsule compared to the parental strain. In contrast, the Δ*rmpD* mutant exhibited no difference in capsule production but lost its hmv phenotype. These results are consistent with some early observations suggesting that capsule overproduction and hypermucoviscosity are probably regulated in separate manners [52].

Of note, not all hv*Kp* isolates display the hmv phenotype, which can result in a negative string test (see below). For instance, a study conducted in China involving 47 *Kp* strains associated with liver abscesses (usually due to hv*Kp*) showed by PCR analyses that all strains carried the virulence genes *rmpA*/*rmpA2*, as well as other virulence genes linked to hypervirulence, such as *iucA* and *iroB* encoding the siderophores aerobactin and salmochelin, respectively (see below). Based on these results, all strains were defined as hv*Kp*, although only 31.9% (15/47) of them exhibited the hmv phenotype by using the string test [53].

### Siderophore Systems for Iron (Fe) Acquisition

Siderophores are small low-molecular-weight chelators that play a crucial role in bacterial Fe acquisition. These molecules are secreted by the bacteria into the external environment, where they bind Fe with extremely high affinity and are then taken back into the bacterial cells *via* specific receptors and transport systems [36]. This process provides the bacteria with the Fe they need to grow and is considered an important VF [18], as it allows the bacteria to survive in the usually Fe-poor environment of the infection site resulting from a process known as nutritional immunity (ie, a series of different Fe-limiting strategies used by the host immune system to protect itself) [54].

Indeed, *Kp* strains with an *in vitro* siderophore production greater than 30 μg/mL have been linked to severe illness and mortality in a mouse systemic infection model [11]. Therefore, this quantitative difference in siderophore production may aid in the discrimination between hv*Kp* and c*Kp* strains, with hv*Kp* exhibiting higher siderophore production levels (see below) [47, 55–58].

*Kp* strains may produce 4 different types of siderophores: aerobactin, salmochelin, yersiniabactin, and enterobactin. Their expression level is linked to the *iuc*, *iro*, *ybt*, and *ent* gene clusters, respectively [11, 18, 59]. In particular, targeting aerobactin and salmochelin is of great importance to recognize hv*Kp* strains.

Aerobactin is expressed in over 90% of hv*Kp* strains and is more specific for this pathotype compared to enterobactin and yersiniabactin, which are often found in both c*Kp* and hv*Kp* [6]. This underscores the critical role of these siderophores as an essential VF in hv*Kp*, particularly in systemic infections [60]. Of note, the genes responsible for aerobactin biosynthesis and transport (*iucABCD* and *iutA* operon) are located on hv*Kp* virulence plasmids (eg, pLVPK and pVir-CR-hvKp4 carry both genes) [36, 61, 62]. Salmochelin, expressed by genes of the *iroA* locus (eg, *iroBCDN*) is also often found and highly expressed in hv*Kp*. Therefore, *iroA* is considered another main VF for hv*Kp* strains [36, 41, 46]. Interestingly, a recent analysis of ~2,500 genomes suggested a high co-occurrence of the *iuc* and *iro* loci in hv*Kp* [63].

In a study by Sheng et al, all *Kp* causing bacteremia and testing positive for the aerobactin encoding gene resulted to be hv*Kp* strains, and well above half of such isolates carried genes coding for salmochelin (86.2%) and yersiniabactin (72.4%) [59]. In another study considering 97 *Kp* genomes of the hypervirulent sequence type (ST) 23, genes encoding all 3 siderophores were found in almost all genomes [64].

### Additional hv*Kp* VFs

In addition to the previously discussed VFs, hv*Kp* may possess further virulence-linked traits such as LPS, colibactin, fimbriae, and *peg-344*.

LPS is composed of lipid A, an oligosaccharide core, and the O antigen. These components, altogether also known as endotoxin, are encoded by *lpx*, *waa*, and *wb* gene clusters, respectively, in all *Kp* strains [36, 46]. LPS functions as a protective barrier against humoral defenses even in the presence of the capsule and also acts as a potent immune activator [11]. Currently, the specific role of LPS produced by hv*Kp* strains in their enhanced virulence compared to c*Kp* remains uncertain [41].

Colibactin is a genotoxic metabolite expressed by genes (*clb*) located on the polyketide synthase genomic island (*pks*), typically found within a chromosomal ICE [18]. The presence of the *pks* island is well-documented in certain strains of *Escherichia coli*, and it has been increasingly recognized in *Kp*, including hv*Kp*. Colibactin induces DNA damage in host cells and appears to contribute to the colonization and pathogenesis of hv*Kp* [11, 27, 46, 59].

Analysis of *Kp* fimbriae has identified two key types: type 1 and type 3 [36]. These fimbriae are encoded by the *fimH* (type 1) and the *mrkABCD* (type 3) gene clusters, and both play crucial roles in biofilm formation [46]. In particular, type 3 fimbriae are recognized as a significant bacterial VF in hv*Kp*, however, their role in hv*Kp* remains poorly understood. Alharbi et al showed that biofilm formation in hv*Kp* isolates was significantly higher than in c*Kp* strains [65, 66]. In contrast, other studies found that hv*Kp* strains were associated with weak biofilm formation [67, 68], showing no significant difference from c*Kp* in this regard [69].

As anticipated above, *peg-344* can be carried by some MGEs in hv*Kp* strains (eg, plasmid pLVPK, but not pVir-CR-hvKp4). It encodes a metabolic transporter with an unknown function located within the inner membrane. When *Kp* is grown in human ascites, the expression of *peg-344* is increased [11, 46]. Additionally, *peg-344* was essential for full virulence in a murine model of pneumonia challenge but did not appear to be related to the induction of sepsis after subcutaneous challenge [70].

### Unusual hv*Kp* Strains

Several authors reported rare hv*Kp* strains lacking the common regulators of the hmv phenotype (*rmpA*/*rmpA2*) or the siderophores [71, 72][73]. For instance, a ST14 MDR *Kp* isolate in Sudan did not possess *rmpA* and *rmpA2*, although it showed an hmv phenotype based on the positive string test. The strain was also of serotype K2 and produced aerobactin and salmochelin, but the hypervirulent phenotype was not analyzed and confirmed with an *in vivo* model [72]. Similarly, 2 MDR-*Kp* strains (ST15 and ST307) from China were reported as hmv and hypervirulent by using the string test or the mucoviscosity assay and the *Galleria mellonella* infection model, respectively. Notably, both strains lacked *rmpA*, *rmpA2,* aerobactin, and salmochelin genes [73]. Overall, it seems that the hmv/hypervirulent phenotypes of hv*Kp* can also be determined by non-classic biomarkers that should be further investigated with appropriate methodologies. In this context, we emphasize that the implementation of the string test or the *G. mellonella in vivo* model may not be appropriate to define the hmv/hypervirulent phenotypes (see below).

## ANTIBIOTIC RESISTANCE

Historically, hv*Kp* strains were typically sensitive to common antibiotic agents, including last-generation cephalosporins and carbapenems. However, in the last years, several concerning reports of the emergence of MDR hv*Kp* strains acquiring different ARGs through HGT of MGEs have been published [32, 35, 64, 74]. In particular, cases of hv*Kp* producing extended-spectrum β-lactamases (ESBLs) and/or carbapenemases have been documented [3, 75].

In this context, there are different mechanisms that can mediate this phenomenon [11, 76]: 1) ESBL-and/or carbapenemase-producing (CP) c*Kp* strains acquire a virulence plasmid (eg, pLVPK) which is typically non-conjugative but can be acquired through co-mobilization by helper conjugative plasmids (usually of IncF type); 2) hv*Kp* strains acquire a conjugative plasmid that carries ESBL and/or carbapenemase encoding *bla* genes; 3) c*Kp* strains acquire both ESBL and/or carbapenemase encoding *bla* genes along with the VFs *via* a virulence hybrid plasmid resulting from the recombination between a resistance and a virulence plasmid [11, 77–83].

### ESBL Producers

Most recent studies focus on the detection and description of CP hvKp strains (see below), while only few performed in the past provide data regarding ESBL-producing hv*Kp* strains. In a survey conducted in China (2013), 37% (85/230) of the *Kp* strains were identified as hv*Kp* based on the presence of *rmpA*. Among them, 13% (11/85) were found to be ESBL producers, with CTX-Ms being the most frequently detected ESBLs [84]. Similarly, Liu et al indicated that 45.7% (80/175) of strains collected in China between 2008 and 2014 were predicted to be hv*Kp* based on the presence of K1 and K2 capsule types or *magA*, *rmpA*, and *rmpA2* genes. Among them, 16.3% (13/80) were ESBL producers [85].

The clinical impact of ESBL-producing hv*Kp* strains is significant, as evidenced by a series of cases involving liver abscess, meningitis, and severe infections [86, 87]. For instance, Xu et al described the first case of endophthalmitis in China associated with a CTX-M-14-producing hv*Kp* strain belonging to clonal complex (CC) 23 and K1 serotype; the *bla*CTX-M-14 gene was acquired through horizontal transmission of a plasmid. The patient's visual activity recovered only after prolonged imipenem treatment [88].

### Carbapenemase Producers

Carbapenem resistance in *Kp* involves various mechanisms, with carbapenemase production being the most significant because of its association to MGEs [76, 89]. This phenomenon is also true for the carbapenem-resistant (CR) hv*Kp* strains [90].

The first CP hv*Kp* strain (belonging to K2 and ST65) was identified in China in 2015 due to the acquisition of a plasmid harboring a *bla*_KPC_ carbapenemase gene [91]. To date, the emergence of CP hv*Kp* strains has been documented worldwide. In this context, the acquisition of a KPC encoding gene is the most common [44, 46, 92–95], but NDM- [29, 96, 97], and OXA-48-like CP strains have also been reported [80, 98].

For instance, Yang et al conducted a screening of 784 KPC-2-producing *Kp* strains in 3 hospitals between 2014 and 2017 in China. They investigated the presence of virulence genes (ie, *rmpA*, *rmpA2*, and *iutA*) revealing that 457 (58%) strains harbored at least 2 of them [99]. In another study from China, 69 non-duplicated CR *Kp* isolates were collected during 2021 to 2022 and analyzed by PCR. As a result, 27 out of 69 (39.1%) strains were considered hv*Kp* based on the presence of *rmpA* and/or *rmpA2* with *iucA*, *iroB*, and *peg-344*. Among these, 22 (81.5%) carried *bla*_K-_ PC-2 and belonged to ST11 [100]. In a study from Germany with CP *Kp* strains collected between 2013 and 2021, the *iuc* gene (aerobactin) was present in 18 of 109 (16.5%). These isolates produced OXA-232 (n=7), OXA-48 (n=6), OXA-48 and NDM (n=3), NDM (n=1), and KPC (n=1) carbapenemases. Seven out of 18 CP hv*Kp* isolates were also resistant to ceftazidime-avibactam, colistin, and/or cefiderocol [80]. In this context, we emphasize that hv*Kp* strains producing KPC variants (eg, KPC-31, KPC-135) conferring resistance to ceftazidime-avibactam are emerging [101, 102].

## EPIDEMIOLOGY

Many studies have identified hv*Kp* strains in clinical samples from humans and animals, but these pathogens are nowadays also reported in contaminated food and the environment [41, 103]. Historically, these studies predominantly originated from Southeastern Asia; however, reports from other areas, including Europe and the United States started to become more frequent in the last 10 years [104–106].

### Infections Due to hv*Kp*

Hv*Kp* strains, particularly MDR hv*Kp*, can cause severe infections and lead to outbreaks with high mortality rates, particularly in immunocompetent individuals [27]. For instance, hv*Kp* bacteremia has a mortality rate up to 37% [107]. In addition, patients with metastatic disease may encounter serious long-term neurological or visual disabilities [108, 109].

Most hv*Kp* strains belong to a relatively small group of clones, indicating the importance of a particular genetic background for the development of hypervirulence. For instance, many studies have shown that strains belonging to the CC23 (particularly the ST23 of capsular serotype K1) and the ST65 and ST86 (both of capsular serotype K2) are frequently associated with hv*Kp* [26, 103, 110]. MDR hv*Kp* strains (especially those producing carbapenemases) are rather linked to other STs, like ST11 (K24/K47/K64), ST15 (K24/K47/K54/K64/K112), ST101 (K62), ST147 (K20/K64), ST231 (K51), ST258 (K106/K107), ST307 (K102), ST395 (K2/K39), and ST512 (K107) [80, 111, 112].

Infections with hv*Kp* are geographically widespread. Initially, hv*Kp* infections were solely documented in the Asia-Pacific region, including China and Singapore [113, 114], with prevalence rates between 12% to 45% during 2008 to 2017 [29–31]. However, at the beginning of the 21^st^ century, there has been a global expansion of hv*Kp* cases, reaching Europe and North America. For example, a study conducted in Spain (2007-2013) found that 53 out of 878 (5.4%) isolates exhibited an hmv phenotype by the string test and belonged to the K1/K2 serotypes, of which 16 (1.8%) being ST23/K1 [26]. Similar trends have been observed in the United States (6.3% in 2009-2010) and Canada (8.2% in 2001-2007) [115, 116]. According to the European Centre for Disease Prevention and Control, most of the contemporary hv*Kp* reported from healthcare facilities in the European Union/European Economic Area countries belong to the ST23/K1 clone [117].

### Gut Colonization with hv*Kp*

With such an increasing incidence of hv*Kp* infections, it is also necessary to focus on the intestinal colonization of hv*Kp*, which can directly precede subsequent infections in healthy people. Numerous studies have confirmed that the human gut is a reservoir for *Kp* [118–120]. However, accurate assessment of hv*Kp* colonization rates in the community is challenging due to the inconsistent use of hv*Kp*-specific markers to distinguish them from c*Kp*. Similar to c*Kp*, colonization by hv*Kp* is likely, but it does not always lead to subsequent infection [11].

Yang et al analyzed data from public databases, revealing that hv*Kp* can indeed colonize the gut of Chinese individuals. They collected 95 gut *Kp* isolates from 69 healthy individuals and found that the carriage rate of hv*Kp* among these individuals was 12% as evidenced by the presence of the *iuc* locus and *rmpA/rmpA2* genes detected by PCR [121]. Observations of hv*Kp* gut colonization have also been reported in other countries. A study among healthy Koreans revealed a hv*Kp* colonization rate of 4.6% in 2007 [122]. In Norway (2015-2016), 5 out of 484 (1%) *Kp* strains isolated from fecal samples were defined as hv*Kp* [123]. Between 2004 and 2010, in Malaysia, Singapore, Taiwan, Japan, and Thailand, colonization rates were 14.1%, 14.9%, 11.3%, 16.7%, and 2.7%, respectively [11, 47].

### Non-human Setting

In a recent report from Egypt, the overall prevalence of hv*Kp* and CR hv*Kp* in diarrheic farm animals was 7.9% and 6.1%, respectively. Of note, hv*Kp* and CR hv*Kp* were detected among all examined farm animal species (cattle, sheep, and goats) [124]. In another study performed in China (2017-2019), the screening of companion animals (dogs and cats) found an overall *Kp* prevalence of 2.0% (n=105). Moreover, 8.6% of the *Kp* strains (n=9) had an hmv phenotype, while 64 isolates (61%) were hv*Kp* based on the *G. mellonella* infection model (see below) [125].

Hv*Kp* have been also isolated from different food and environmental sources. In a study from China (2017), 2 ST23 hv*Kp* carrying *bla*_KPC-2_ were isolated from cucumbers in ready-to-eat vegetable samples [126]. Li et al (China) reported the presence of the tigecycline resistance gene *tet(X4)* in hv*Kp* strains from pork samples collected from multiple markets in 2020 [127]. Three MDR hv*Kp* strains were isolated from public water environments in Brazil (2018), highlighting another potential source of hv*Kp* acquisition [128]. In Egypt, CP hv*Kp* strains have been isolated from fresh oysters [129]. These data support the need for a One Health approach, including surveillance of non-human settings, to reduce the spread of hv*Kp*.

## DETECTION TESTS

For many years, infections due to hv*Kp* have been diagnosed solely on the basis of clinical presentation [11]. Meanwhile, several phenotypic and genotypic diagnostic tests have been developed in an attempt to specifically detect hv*Kp,* which vary widely in methodology and accuracy (Table 2).

**Table 2.** Available Methods for the Assessment of the Pathotype of *K. pneumoniae*: Main Advantages and Disadvantages

Detection tests	Method	Advantages	Disadvantages
***In vivo* models**	Mouse killing assay	High accuracy	Complex operation High cost Time-consuming Ethical concerns
	*G. mellonella* infection model	Cost-effective No ethical approval No dedicated facility	Cannot accurately differentiate hv*Kp* from c*Kp*
**Phenotypic tests**	String test	Low cost Easy to use Short detection time	Low sensitivity and specificity
	Siderophore production tests	Low cost	Time-consuming Not accurate for MDR strains
	Mucoviscosity assay	Low cost Accurate (including MDR strains)	Time-consuming
	Serum killing assay	Low cost Easy to perform	Accurately identifies only K1 and K2 serotypes
	Tellurite resistance test	Low cost Easy to use	Relatively low specificity
**Non-phenotypic tests**	Multiplex qPCR	Highly sensitive and specific Detection of multiple targets in a single reaction	Limited to designed genetic targets Non-standardized if designed in-house
	LAMP (Eazyplex^®^ hv*Kp* assay)	Easy to use Short detection time Accurate	Relatively high cost
	RAA	High sensitivity and specificity Detection of multiple targets	Complex assay design Limited to designed genetic targets Non-standardized if in-house designed
	MALDI-TOF MS	Short detection time Low cost	Sample preparation is time-consuming It distinguishes only serotype K1 *vs*. non-K1
	ICT (ICS)	Easy to use Short detection time	It distinguishes only serotype K1 and K2
	Raman spectroscopy	Short detection time Low cost per reaction/analysis Detection of multiple targets Accurate	High cost for apparatus Data processing and analysis challenges Not yet standardized
	NGS Illumina	High accuracy High throughput Cost-effective (lower cost per base)	Short reads (difficult to resolve large genomic elements such as plasmids) Long library preparation (affecting TATs) Limited real-time analysis
	NGS Oxford Nanopore/PacBio	Long-reads (aiding in the assembly of full genomes) Real-time sequencing (Nanopore) Minimal sample preparation Available PCR-free library preps High consensus accuracy (PacBio)	Lower throughput Lower read accuracy *vs*. Illumina Higher cost per base Data processing and analysis challenges
	mNGS	Ability to analyze genetic diversity of the strains directly from clinical samples Can be used for real-time surveillance (Nanopore) of hv*Kp* outbreaks Supplement culture-based methods	Higher costs per base (simultaneous organisms are sequenced) Special gDNA isolation kits Data processing and analysis challenges

**Note.** Multiplex qPCR, multiplex quantitative polymerase chain reaction; LAMP, loop-mediated isothermal amplification; RAA, recombinase-aided amplification; MALDI-TOF MS, matrix-assisted laser desorption/ionization time-of-flight mass spectrometry; ICT, immunochromatographic test; ICS, immunochromatographic strip; NGS, next-generation sequencing technologies; TAT, turnaround time; mNGS, metagenomics NGS; *Kp*, *K. pneumoniae*; hv*Kp*, hypervirulent *K. pneumoniae*; c*Kp*, classical *K. pneumoniae*.

We emphasize that a consensus definition of hv*Kp* does not yet exist. This controversial issue affects the interpretation of epidemiological data (see above) and also the comparison of the analytic performance of diagnostic tests described below [56, 106]. In this context, we believe that accurate and powerful studies combining whole-genome sequencing analysis of key biomarkers (eg, *iucA*, *rmpA*, *rmpA2*, *iroB*, *peg-344*) and *in vivo* murine testing (eg, Russo et al [33]) will provide essential insights to elaborate a standardized definition for hv*Kp*.

### *In vivo* Models

**Mouse killing assay.** The mouse lethality assay is an accurate method for identifying hvKp and differentiating it from cKp strains. Despite its accuracy, the assay is time-consuming, costly, and complex, limiting its widespread application (Table 2). However, mouse infection models remain a standard for assessing pathogen virulence, including that for Kp. Typically, the lethal dose (LD) for hvKp in a mouse model is less than 106 colony forming units (CFUs), while for cKp it exceeds 107 CFU [130]. The assay can be performed using either intraperitoneal (IP) or subcutaneous (SC) injection methods.

In one study, mice were infected with 100 µL of varying concentrations (10^3^ to 10^6^ CFU) from six hv*Kp* strains. Mortality was monitored over 7 days. The 50% lethal dose (LD_50_) for 3 strains (2 K2 and 1 K1) was found to be less than 10^2^-10^3^ CFU, while the remaining 3 strains (K57, K105, and another K1) had an LD_50_ of 10^4^-10^5^ CFU. The LD_50_ for K1 strains was 10^2^-10^5^ CFU, while for K2 strains it was 10^2^-10^3^ CFU [53]. Wang et al conducted a study showing significant variation in LD_50_ among hv*Kp* strains. They injected mice with 100 µL of bacterial suspension intraperitoneally and observed mortality for 14 days. Mice challenged with K1/K2 isolates showed an LD_50_ ranging from ≤10^2^ to 2x10^3^ CFU, whereas mice infected with ST11 isolates with serotypes K20, K47, and K64 exhibited no illness symptoms with a high LD_50_ of >10^7^ CFU [131].

Russo et al found variability in virulence among hv*Kp* strains [57]. After SC challenges of CD1 mice, the strains were categorized based on the lethal challenge inoculum (CI) as follows: fully virulent hv*Kp* (_fv_hv*Kp*) strains that were lethal at a CI ≤10³ CFU, partially virulent hv*Kp* (_pv_hv*Kp*) strains that were lethal at a CI between 10^4^ and 10^7^ CFU, and c*Kp* strains that were not lethal even at a CI of 10^7^ CFU. Mortality rates for _fv_hv*Kp* after SC challenges were 80% at 10^2^ CFU and 100% at 10^3^ CFU, compared to _pv_hv*Kp*, which showed 0% mortality at both 10^2^ and 10^3^ CFU. However, at CIs of 10^4^ to 10^7^ CFU, _pv_hv*Kp* mortality rates were 20%, 70%, 80%, and 80%, respectively. For IP challenges in CD1 mice, mortality rates for _fv_hv*Kp* were 40% at 10^2^ CFU and 60% at 10^3^ CFU. In contrast, challenge with an inoculum of 10^4^ CFU of _pv_hv*Kp* showed 0% mortality in 2 mouse types (ie, BALB/c and CD1) and 50% mortality in 1 (C57BL/6), indicating that IP challenges of BALB/c and CD1 mice, but not C57BL/6 mice, may be used to clearly differentiate _fv_hv*Kp* from pvhv*Kp*. In conclusion, both SC and IP challenge models effectively distinguished between hv*Kp* and c*Kp* strains. Of note, no mortality was observed in SC challenges with c*Kp* challenge inoculum up to 10^8^ CFU, while IP challenges with c*Kp* had an LD_50_ >10^7^ CFU [57].

In another study, mice were injected subcutaneously with concentrations ranging from 2x10^3^ to 5x10^3^ CFU for both hv*Kp* and c*Kp* strains and with CI of 3x10^7^ to 6x10^7^ CFU for c*Kp* strains, monitored over 14 days. The mean 14-day death rates for hv*Kp* and c*Kp* strains were 91.2% and 0% with CI of 2x10^3^ to 5x10^3^ CFU, respectively. The mortality rate for the c*Kp* strain remained at 0% even with 4-log higher CIs of 3x10^7^ to 6x10^7^ CFU [132].

***Galleria mellonella* infection model.** The *G. mellonella* infection model has been employed to investigate various bacteria and assess the virulence of *Kp* due to the ease and cost-effectiveness of obtaining larvae (Table 2) [9]. Typically, 10µL to 20µL of a specific concentration of *Kp* is injected into *G. mellonella* larvae through the left second hind proleg, which are then maintained at 37°C in darkness. Larval survival is monitored daily, and death is determined when larvae cease responding to mechanical stimuli and exhibit a change in body color from yellowish to black (ie, melanization), with the time of death recorded [9].

Li et al demonstrated that using the *G. mellonella* infection model alone for identifying hv*Kp* yielded a sensitivity of 97.8% and a negative predictive value (NPV) of 95.2%. However, the specificity and positive predictive value (PPV) were notably lower at 34.5% and 53.6%, respectively. Combining the *G. mellonella* infection model with the string test led to a significantly improved sensitivity, specificity, PPV, and NPV (95.6%, 94.8%, 93.5%, and 96.5%, respectively) [130].

The use of *G. mellonella* to differentiate hv*Kp* from c*Kp* was also investigated in other studies. Russo et al found that when a CI of 1x10^5^ CFU was administered, the 5-day mortality rates for hv*Kp* and c*Kp* strains were 93.4% and 71%, respectively. However, for CI ranging from 1x10^4^ to 5x10^4^ CFU and 1x10^5^ to 5x10^5^ CFU, the differences in mortality were minimal (25.8% and 22.4%, respectively). This indicates that the *G. mellonella* model alone may not be a reliable method for differentiating hv*Kp* from c*Kp* [132].

### Phenotypic Tests

**Figure 1.**
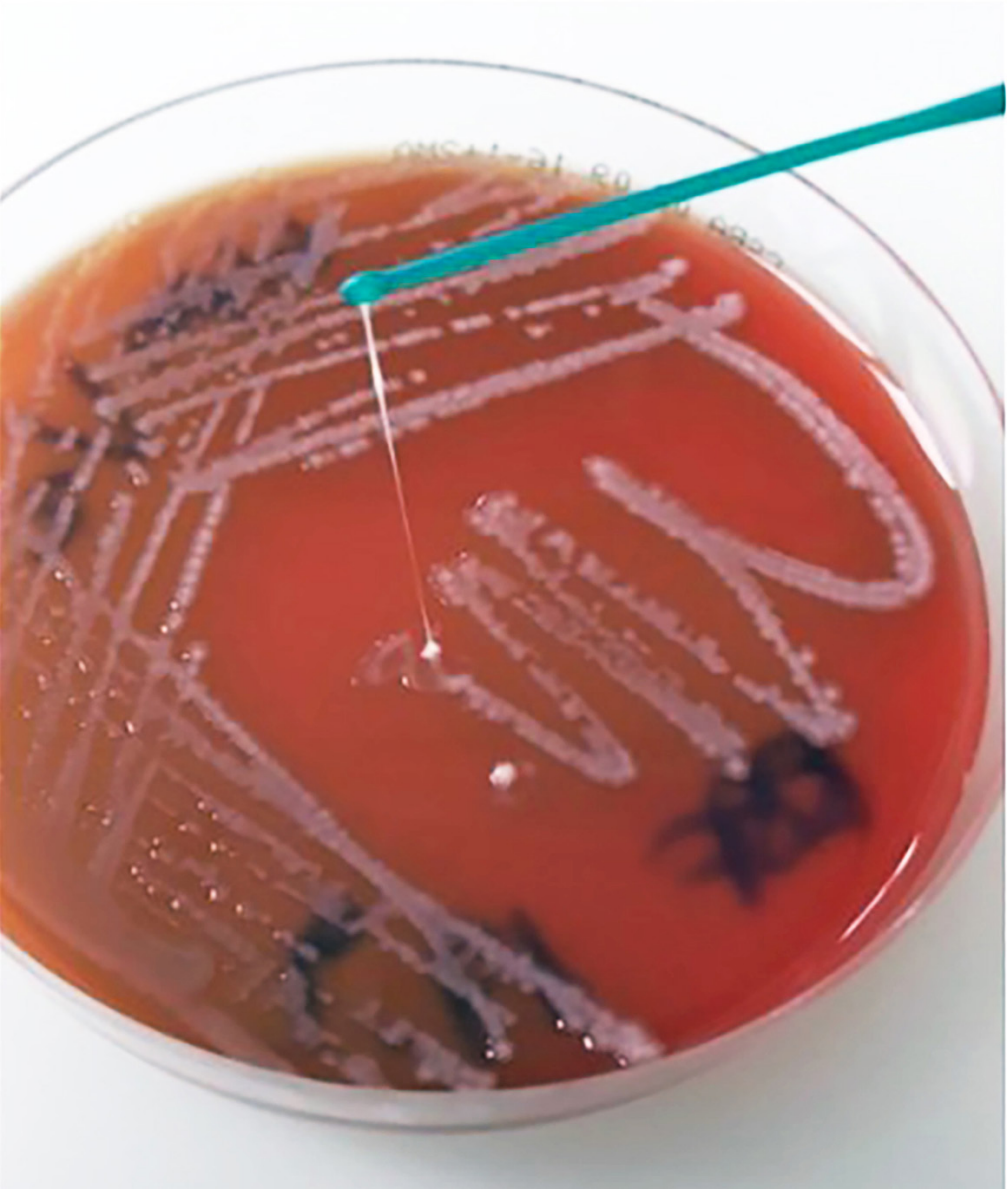
**String test on Columbia agar with 5% sheep blood (CSBA, Oxoid).** Since a string with a length >5 mm was observed, the test result was considered positive (ie, hypermucoviscous phenotype of the *K. pneumoniae* strain).

**String test.** This test is classically used to determine the hmv phenotype of *Kp*, a trait that typically characterizes most hv*Kp.* This simple test involves touching a bacterial colony on a blood agar plate with an inoculation loop and then pulling the loop away (Figure 1) [1, 9]. A positive result is indicated by the formation of a viscous string stretching from the bacterial colony to the loop measuring ≥5 mm [15, 43]. However, recent research indicates that the string test, which is still widely used as the primary assay to identify hv*Kp* strains, is less effective than other methods.

For instance, Russo et al found that the string test resulted in an accuracy, sensitivity, and specificity of 90%, 89%, and 91%, respectively, which were inferior to the testing values obtained for several other genotypic biomarkers (ie, *peg-344*, *iroB*, *iucA*, *rmpA*, and *rmpA2*) [55]. In another study, the positive rate of the string test was only 13.8%, which was equal to or lower than the positive detection rates obtained by PCR analysis individually targeting the 5 major virulence genes *peg-344*, *iroB*, *iucA*, *rmpA*, *rmpA2* (ie, 43.6%, 19.2%, 50%, 10.6%, and 50%, respectively) [133]. In a further study involving 56 strains, previously identified as hv*Kp* based on the clinical presentation (ie, from patients with liver abscesses), only 64.3% (n=36) exhibited a positive hmv phenotype by string test. Among these 36 hmv strains, 100% were positive for the *rmpA* gene by PCR. However, 95% of the non-hmv hv*Kp* strains were also positive for *rmpA*, suggesting that this gene may be necessary, but not sufficient for the development of the hmv trait [134].

In conclusion, reliance on the string test alone appears inadequate for the identification of hv*Kp* due to its suboptimal sensitivity and specificity (ie, negative and positive string test results may also be reported for hv*Kp* and c*Kp* strains, respectively) (Table 2) [5, 135–137].

**Siderophore tests.** Assessing siderophore production shows promise for differentiating hv*Kp* from c*Kp* strains. Both qualitative and quantitative methods could be used to evaluate siderophore production by the bacterial strains (Table 2) [33].

In the qualitative siderophore production test, strains are grown overnight at 37°C in M9 iron-chelated medium containing casamino acids (c-M9-CA). After centrifugation, the supernatants are diluted 5-fold in c-M9-CA. These are then mixed with a 98% chrome azurol S (CAS)-containing siderophore assay solution and incubated in the wells of a flat-bottomed 96-well plate in the dark for 30 minutes. A color change from blue to orange indicates the production of siderophores, signifying a positive test result. Russo et al employed this method to analyze and differentiate between c*Kp* and hv*Kp* strains, achieving accuracy, sensitivity, and specificity of 93%, 91%, and 96%, respectively [55].

The quantitative siderophore production test follows the same initial steps as the qualitative test. However, after incubation of the reaction mixture in the 96-well plate for 30 minutes in the dark, the results are read at 630 nm with a spectrophotometer, using a standard curve to quantify the siderophore concentration for each sample [138]. Although initially promising, a recent study by Russo et al has shown that the quantitative siderophore production was similar for ESBL-/CP hv*Kp* and c*Kp* (135.8 µg/mL *vs*. 139.8 µg/mL, respectively), indicating that: a) the test cannot reliably distinguish between contemporary hv*Kp* and c*Kp* strains, and b) it may be unsuitable for routine clinical laboratory use [33].

**Mucoviscosity assay.** Based on previous findings, the evaluation of mucoviscosity could also be used to distinguish between hv*Kp* and c*Kp* strains, given the stronger association of the hmv phenotype with the hypervirulent pathotype [139]. To assess mucoviscosity, according to Russo et al, cultures are grown in either Luria-Bertani (LB) or iron-chelated M9 minimum medium supplemented with trace elements (5 μg/mL CaCl_2_, 1 μg/mL CoCl_2_, 20 μg/mL MgCl_2_, 10 μg/mL MnCl_2_) (c-M9-CA-te) for 24 hours at 37°C. The OD_600_ is standardized to 1.0 using either LB or c-M9-te minimal medium (pre-spin OD_600_). After centrifugation of the OD_600_-adjusted cultures, 700 μL of supernatant are used for OD_600_ measurement (post-spin OD_600_). Mucoviscosity is recorded as the ratio of post-spin to pre-spin OD_600_ [33].

In their study, Russo et al found that in contrast to the quantitative siderophore production test, the mucoviscosity test was able to discriminate between ESBL-/CP hv*Kp* and c*Kp* (ie, the hv*Kp* exhibited significantly higher mucoviscosity compared to c*Kp* when grown in LB medium (*P*=0.0113) and in c-M9-CA-te medium (*P*< 0.0001)) [33].

**Serum killing assay.** Numerous studies have investigated the bactericidal activity of normal human serum against various Gram-negative bacteria [140], demonstrating minimal impact on some *Kp* strains [141]. Notably, the resistance to human serum could be a crucial VF.

According to Gao et al, 25 μL of bacterial suspension prepared from the mid-log phase is mixed with 75 μL of human serum and then incubated at 37°C for 3 hours. The response to serum killing is assessed in terms of viable cell counts and is graded on a scale from 1 to 6, where grades 1 to 2 represent high sensitivity, grades 3 to 4 indicate intermediate sensitivity, and grades 5 to 6 indicate resistance. Their findings revealed that 46.8% (22/47) of hv*Kp* strains (all carrying *rmpA*/*rmpA2* genes) demonstrated resistance to serum killing, with K1/K2 serotype strains exhibiting higher resistance compared to other serotypes [53]. Similarly, Wang et al corroborated this observation, indicating that K1/K2 serotypes displayed a higher resistance with grades 5 to 6 [131]. These results suggest that serum killing tests could be particularly useful for identifying hv*Kp* of the K1/K2 types (Table 2).

**Tellurite resistance test.** Tellurite resistance in *Kp* is strongly associated with hv*Kp* strains. The tellurite resistance gene (*terW*) is linked to the virulence of hv*Kp* and is often found on the virulence plasmid pLVPK carried by hv*Kp* [142]. However, it is not exclusive to hv*Kp* [46, 55]. The tellurite resistance test is considered simple and easy. When strains form black colonies on selective media containing tellurite, they are presumptively considered to be hv*Kp* strains [9].

Sanikhani et al screened a total of 477 non-repetitive *Kp* strains isolated from 2 educational hospitals in Iran between 2019 and 2020 using the tellurite resistance test. They found that 34.2% (163/477) of the isolates were tellurite resistant, and among these, 102 (62.6%) were defined as hv*Kp* isolates based on PCR positivity for at least one among *iucA*, *iutA*, or *peg-344* targets [143]. Furthermore, Wu et al established a MacConkey agar medium containing 4 μg/mL potassium tellurite to detect tellurite resistance. Testing a collection of c*Kp* and hv*Kp* strains (of which 13.3% and 70.6% positive for *terW*, respectively), this method showed accuracy, sensitivity and specificity of 94.9%, 92.7% and 100%, respectively. These results indicate that this test may be a promising adjuvant strategy to distinguish c*Kp* from hv*Kp* (Table 2). However, further confirmatory studies should be performed because presence of *terW* may not correspond to phenotypic tellurite resistance [144].

### Non-phenotypic Tests

**Multiplex quantitative PCR (multiplex qPCR).** Quantitative PCR (qPCR) consists of the PCR amplification of a single or multiple target genes coupled with the quantitative detection of the exponentially amplified DNA product(s) by various methods, such as fluorescence emission with SYBR Green or TaqMan probes. It has emerged as a powerful tool in microbial diagnostics due to its high sensitivity, specificity, and rapidity of results [145, 146].

Xu et al established and developed a rapid quadruple qPCR assay that includes, in a single reaction, a positive control specific for *Kp* (*gltA*), 3 hv*Kp*-specific markers (*iucA*, *rmpA*, and *rmpA2*), and 1 carbapenemase target (*bla*_KPC_) [15]. To validate the assay, a total of 84 *Kp*-containing clinical samples were tested showing that 31 had hv*Kp* and 53 had c*Kp*. Based on the outcomes of conventional PCR, it was confirmed that in the 31 hv*Kp* the *iucA* gene was present in all strains, while other genes were distributed as follows: *rmpA* (n=20), *rmpA2* (n=18), *iroB* (n=17), and *peg-344* (n=1). Various combinations of these genes were also observed, with 8 out of 31 hv*Kp* and 1 out of 53 c*Kp* carrying *bla*_KPC_. From the clinical samples, 67 strains were successfully isolated and tested using the string test, resulting in 20 strains testing positive (hv*Kp*) and 47 testing negative (c*Kp*). There were 26 discrepant samples between the multiplex qPCR and the string test: 16 strains were identified as hv*Kp* only by qPCR, and 10 strains were identified as hv*Kp* only by the string test. Notably, in the 10 strains identified as hv*Kp* by the string test, no VFs were detected by conventional PCR. To evaluate the accuracy of the multiplex qPCR, an outbred murine infection model was used (10^7^ CFU inoculum), showing ~80% mortality rates in strains identified as hv*Kp* by qPCR, while no deaths were observed in strains identified as c*Kp*. These findings indicate that only the multiplex qRT-PCR assay can accurately distinguish hv*Kp* from c*Kp* strains (Table 2).

**Loopmediated isothermal amplification (LAMP).** The LAMP technique allows for the amplification and fluorescent detection of the target DNA at a constant temperature. Remarkably, a typical genomic extraction is not strictly required, as this highly sensitive method takes advantage of the strand displacement activity of the *Bst* DNA polymerase, which is robust to amplification inhibitors [147, 148].

In 2020, Liao et al designed a LAMP detection method for hv*Kp* utilizing *peg-344* as the target molecular biomarker. This approach achieved a sensitivity 100 times greater than that of conventional PCR to detect hv*Kp* isolated from human blood samples. Their findings underscore the potential of LAMP technology for rapid molecular diagnostics of hv*Kp*, given its affordability and simplicity of use [149]. However, we should emphasize that *peg-344* is not always carried by hv*Kp* strains (eg, the pLVPK virulence plasmid carries it, but not its variant pVir-CR-HvKp4) [11]. Therefore, the LAMP approach should target additional virulence genes.

The Eazyplex^®^ Superbug CRE assay (Amplex Diagnostics, GmbH) is a commercial rapid LAMP assay designed to identify carbapenemases in *Enterobacteriaceae*, including *Klebsiella* spp. [150]. In combination with this test, Rödel et al aimed to investigate the performance of the Eazyplex^®^ hv*Kp* assay (Research-Use-Only) that specifically detects virulence genes in hv*Kp*. This assay contains lyophilized master mixes featuring primers targeting *rmpA/A2*, *iucC*, *iroC*, *ybt*, and *clb*[151]. Their findings revealed that 14 out of 87 isolates from invasive infections (16.1%) harbored at least one of the virulence genes, with an increased Kleborate virulence score of ≥2 (see below). Among these, nine scored 4 or 5 (10.3%). The hypermucoviscosity assessed by the string test was positive for 7 of the 14 isolates, while *rmpA/A2* were detected in 9 isolates. Of note, the time from test to result for this assay was less than 15 minutes, indicating that this assay can represent a valuable option for identifying hv*Kp*, especially if used in combination with the Eazyplex^®^ Superbug CRE assay to detect potential CR hv*Kp* (Table 2) [151].

**Recombinaseaided amplification (RAA).** RAA is an isothermal amplification technique based on recombinase-aided polymerase amplification, known for its high sensitivity and specificity [9].

Yan et al established a rapid and convenient diagnostic tool for identifying infections caused by hv*Kp*. The novel RAA assay targeting *peg-344* and *rmpA* was validated on 208 clinical samples, including 158 *Kp*-positive samples collected from healthy individuals (n=60) and inpatients with pneumonia (n=80), bloodstream infection or liver abscess (n=68), demonstrating 100% sensitivity and specificity compared to qPCR by detecting all hv*Kp*-positive samples, but owning a fivefold lower limit of detection [48].

**Matrix-assisted laser desorption/ionization time-of-flight mass spectrometry (MALDI-TOF MS).** MALDI-TOF MS is a rapid technique to discriminate unique protein signatures based on mass spectrometry [9].

In 2015, Huang et al developed a MALDI-TOF MS method for the detection and differentiation of K1 and non-K1 *Kp* strains with an accuracy of 94.1% and 90%, respectively [152]. This method is currently limited by the fact that it cannot distinguish hv*Kp* with other capsule polysaccharide types. Therefore, it needs further improvement, but it could still be applied alongside other conventional genotyping techniques for its rapidity and accuracy (Table 2).

**Immunochromatographic strip.** Antigen detection by using immunochromatographic tests (ICTs) can be used to detect components of bacteria [153]. The ICTs are often lateral flow immune assays where antigen(s) recognition is assured by a monoclonal antibody (mAb) specific to the target analyte labelled with a visual tag. Results are simply interpreted by visualization of a colored line in the test pad. These ICTs are very useful because of their short execution time, low cost, accuracy, lack of additional instrumentation, ease of implementation and minimal hands-on time [153, 154].

Since the *Kp* serotypes K1 and K2 are primarily associated with community-acquired infections and bacteremia, and also often presenting the hv*Kp* pathotype, Siu et al developed a colloidal-based immunochromatographic strip (ICS). This ICS incorporates anti-*Kp* capsular polysaccharide polyclonal antibodies and is designed to detect *Kp* serotypes K1 and K2 [155]. The ICS offers a rapid and straightforward detection method, providing results within 5 minutes after loading the samples obtained by placing diluted cultures onto the ICS. The assay was validated by testing 100 clinical isolates that had been collected from previous studies, including 30, 20, and 50 isolates of *Kp* serotypes K1, K2, and non-K1/K2, respectively. Notably, there were no false-positive or false-negative results observed with the ICS, when compared to the results obtained by conventional PCR and serum agglutination assay, indicating a high degree of sensitivity and specificity [155].

Wang et al aimed to further evaluate the efficacy of this ICS assay for detecting *Kp* serotypes K1 and K2 in pus samples from liver abscess and in positive blood culture samples [156]. Among the 108 *Kp* samples, ICS testing of blood culture samples identified the presence of 14 serotype K1 and 16 serotype K2 *Kp*. However, single colony testing revealed that 2 non-K1/K2 results were erroneously identified as serotype K2. PCR typing of these 2 isolates with discrepant results showed that both were of the K5 serotype [156].

As for the above MALDI-TOF MS approach, the ICS is currently limited by the fact that it can only detect *Kp* serotypes K1 and K2, and not specifically the hv*Kp* pathotype (Table 2). However, this assay could be a valuable addition to current diagnostic methods for its rapidity and convenience.

**Raman spectroscopy.** The Raman spectroscopy is a rapid, low-cost, and highly-sensitive analytical technique where a scattered light is used to measure the vibrational energy modes of a sample to identify its chemical constituents. Specifically, this method can be used to identify bacteria and their ARGs or virulence genes [157]. For instance, Lu et al developed a convolutional neural network (machine learning algorithm) to interpret Raman spectra from 71 *Kp* strains showing good accuracy in identifying carbapenemase/mobile colistin resistance genes along with *rmpA*/*rmpA2* [158]. More recently, several studies have shown the potency of Raman spectroscopy coupled by machine learning to identify hmv *Kp* strains. In this way, Fernandez-Manteca et al were able to identify with high accuracy (94%) the K1, K2, K54, and K57 capsular serotypes in 20 hmv *Kp* strains, also distinguishing 6 non-mucoid isolates [159]. In another study, Zhang et al differentiated 10 hmv from 10 c*Kp* strains with 99% accuracy [160].

Overall, the Raman spectroscopy combined with artificial intelligence represents a very promising technique to rapidly identify hv*Kp* strains. However, this technology remains distant from being implemented in clinical laboratories, with its application currently confined to research settings (Table 2).

### Sequencing Approaches

The detection of *Kp* together with its pathotype and ARGs is crucial for the timely implementation of appropriate clinical interventions and infection control and prevention measures, particularly to promptly identify MDR hv*Kp*. Due to the limitations of current detection methods in this regard (see above), there is an urgent need to develop new and more efficient diagnostic tools.

**Next-generation sequencing (NGS) technologies.** NGS provides a comprehensive, high-throughput approach to microbial genomic analysis by sequencing millions of DNA fragments at the same time, allowing a full characterization of the pathogen, including the identification of VFs and ARGs. Various NGS techniques, generating short (eg, Illumina and Ion Torrent) or long reads (eg, PacBio and Oxford Nanopore technology (ONT)) have been used for this scope (Table 2) [161].

In particular, there are many studies that have used short-read only NGS (short-NGS) for epidemiologic investigations, especially for genomic confirmation and characterization of hv*Kp* isolates. For example, short-NGS has been implemented extensively to gain insight into hv*Kp* suspect isolates (eg, string-test positive) from clinical specimens (eg, liver abscess) derived from retrospective studies, thereby further elucidating potential genomic mechanisms linked to the virulence phenotype (ie, presence of VFs) [99, 162–166]. However, despite the successful use of short-NGS to characterize hv*Kp* isolates, it is well known that short-NGS may not accurately elucidate important genomic elements (eg, _p_*rmpA*, _p_*rmpA2*, and *peg-344*) that are typically localized in MGEs (ie, virulence plasmids). To overcome this problem, some studies have opted for long-read only NGS (long-NGS) to generate complete genomes of hv*Kp* isolates using the ONT or PacBio platforms [167, 168]. Lastly, long-NGS using ONT, may be used to directly determine characteristic epigenetic signatures of hv*Kp*. Ghosh et al showed that hv*Kp* strains possess a significantly higher levels of methylation in chromosomal DNA and extrachromosomal elements compared to c*Kp*; this hypermethylation (ie, GATC and CCWGG motifs) was particularly rich in the virulome rather than in genes not directly associated with virulence [169].

As discussed above, long-NGS allows for complete genome characterization of hv*Kp*, making it an ideal choice for epidemiologic studies. In this context, it is worth noting that the MinION offered by ONT is a small portable sequencing device that allows the generation of long-reads generally in the range of >10 kb, thus capable of resolving structural variations, long repeat regions, and genomic copy-number alterations, requiring only a very little upfront capital investment [170].

Although PacBio reads have historically been more accurate (>99.9%) than ONT reads, newer basecalling models (eg, super accuracy (SUP) basecalling) allow the current Nanopore R10.4 technology (V14 chemistry) to offer comparable performance (>99.1%) [171, 172]. In addition, the availability of newer assembly polishing tools (eg, Medaka) further increases the usability of ONT data for single nucleotide polymorphism (SNP) studies.

For instance, the study by Foster-Nyarko et al utilized sequencing data obtained from 54 unique *Kp* strains to evaluate the efficacy and accuracy of ONT assemblies in identifying ARGs, STs, and VFs compared with short-NGS and hybrid methods [173]. The authors found that the implementation of the R9.4.1 flow cell generated basecalled data, implementing the Fast, High Accuracy, and SUP models, along with polishing assemblies with Medaka, produced high-quality assemblies for determining MLST, K/O locus type, VFs, and ARGs. However, the quality of such ONT assemblies was still not sufficient for SNP analyses (eg, outbreak investigations) compared to Illumina-only assemblies [173]. Therefore, higher-quality data generated by either newer Nanopore V14 chemistry or PacBio technology may be ideal for high-resolution *Kp* studies at the SNP level.

A more established approach is to combine both short-NGS and long-NGS to generate hybrid genome assemblies, appropriate for high-resolution SNP analyses, with the goal to accurately characterize and link ARGs and VFs to their corresponding genomic elements (ie, chromosome and plasmids) [174]. In this context, the implementation of hybrid assemblies has been important in large retrospective epidemiologic investigations of hv*Kp* clinical isolates necessary to elucidate high-risk clones, ARGs, VFs, and associated genomic elements [4, 175–177]. Similarly, such high-resolution studies are also important for the characterization of MDR hv*Kp*, especially those possessing carbapenemases, which may be plasmid-associated and thus of critical epidemiological importance [176, 178–180].

Regardless of the sequencing approach implemented, *in silico* screening for *Kp* VFs can be performed using specialized bioinformatics tools. For example, one of the best-known tools is Kleborate, which allows the user to detect VFs in a given genome assembly by assessing their presence in ICE*Kp* (*ybt*, *clb*, *iro*, *rmp*) and virulence plasmids (*iro*, *iuc*, *rmpA*, *rmpA2*) [181]. In particular, Kleborate determines a virulence score ranging from 0 to 5 (ie, aerobactin, 3 points; colibactin, 1 point; yersiniabactin, 1 point), with a score of 3 or higher indicating significant VFs, which may help researchers differentiate hv*Kp* from c*Kp* at the genomic level, but not for predicting the hypervirulent pathotype [33, 163]. Lastly, Kleborate also provides further information such as *Klebsiella* spp. identification, ST and ARGs (including SNPs), K (capsule) and O antigen (LPS) serotype prediction (implementing Kaptive [182]), making it a powerful tool for *Kp* genomic investigations.

Other platforms, such as Pathogenwatch (https://pathogen.watch/) and Institute Pasteur (https://bigsdb.pasteur.fr/), both incorporating Kleborate, allow researchers to screen genome assemblies for *Kp* VFs. Finally, there are other (non-Kleborate-based) options for researchers to screen for VFs, such as AMRFinder, which implements the NCBI reference gene catalog database (https://www.ncbi.nlm.nih.gov/pathogens/refgene/), and the comprehensive virulence factor database (VFDB; http://www.mgc.ac.cn/VFs/) [183, 184], which mostly requires manual implementation, but can be used by other tools such as ABRicate (https://github.com/tseemann/abricate).

**Metagenomics approaches.** Metagenomic NGS (mNGS; or shotgun-metagenomic sequencing) is valuable for understanding the genetic diversity of a population of strains by analyzing capsular serotypes, identifying ARGs and virulence-associated genes directly from clinical specimens, unlike methods relying on traditional cultivation methods [161]. In this context, very few studies have implemented mNGS, specifically Illumina-based, to characterize hv*Kp* directly from clinical specimens.

Liu et al detected a total of 30 metagenome reconstructed *Kp* strains implementing PanPhlAn (and companion StrainPhlAn) from 150 clinical specimens (the majority were sputa and bronchoalveolar lavage fluids). As a result, the authors identified 399 virulence-associated genes, showcasing the power of metagenomic sequencing to resolve *Kp* at the strain level and demonstrating the genetic diversity of virulence determinants found, including the *ybt* locus (yersiniabactin), *iucABCD* and *iutA* (aerobactin), *iroBCDE* and *iroN* (salmochelin), and the capsule production-associated genes (*cpsA* and *rmpA*) [185].

Case studies have also validated the use of mNGS to identify hv*Kp* directly in clinical samples. For instance, in a study by Peng et al, one hv*Kp* strain belonging to ST23-K1 with *iutA* and *rmpA* genes was successfully identified from bronchoalveolar lavage fluid [186]. Another case study by Xie et al rapidly identified one hv*Kp* harboring *rmpA*/*rmpA2* and *iutA* genes from a liver abscess drainage fluid sample [187]. Moreover, other exceptional cases have applied mNGS to identify hv*Kp* concurrently or faster than culture-based methods [188, 189]. Finally, a case of the implementation of ONT-based mNGS was reported, which allowed the accurate and rapid identification (5-7 hours) of a hv*Kp* in liver abscess fluid [190].

Therefore, the implementation of mNGS for the detection of hv*Kp* from specimens obtained from a suspected site of infection is critical for clinical management, as it provides rapid and accurate pathogen detection, enabling personalized patient treatment strategies by overcoming the limitations of traditional culture-based methods (Table 2) [191].

## CONCLUSIONS

The prevalence of hv*Kp* strains has increased globally, posing a significant public health challenge worldwide. MDR hv*Kp* strains (especially those producing ESBLs and/or carbapenemases) require rapid and accurate detection for effective clinical management. However, there are currently no agreed-upon biomarkers for this pathotype definition. Therefore, current conventional diagnostic methods, although simple to perform, still have many disadvantages, such as a limited specificity and sensitivity, resulting in an inability to identify hv*Kp* strains with complete accuracy.

*In vivo* models are generally more accurate in assessing pathogen virulence. In this context, confirmation of the hypervirulent phenotype of *Kp* strains in the mouse model is crucial, especially when evaluating the accuracy of new tests developed to specifically detect hv*Kp*. However, mice studies are time- and resource-consuming, while recent studies have highlighted the limitations of using the cheaper *G. mellonella* infection model as a reliable method to distinguish between hv*Kp* and c*Kp* strains. In contrast, other molecular *in vitro* methods, such as qPCR or LAMP-based tests, may exhibit superior sensitivity and specificity, especially when the exact biomarker targets used to define hv*Kp* will be formally set.

Overall, there is an urgent need to develop new and rapid diagnostic methods in this field. NGS approaches, including technologies like ONT, able to simultaneously identify virulence genes and ARGs, have shown a great potential to become powerful tools for comprehensive pathogen characterization, but they still rely mostly on traditional culture isolation, affecting sensitivity and time to result (turnaround time). Conversely, methods like mNGS offer a promising avenue for molecular characterization directly from clinical specimens. Future advances in this area hold great promise for significantly improving diagnostic accuracy, guiding more effective treatment strategies, and improving infection control measures in clinical settings to prevent further large-scale spread of hv*Kp*.
